# Outcome of Facial Burn Injuries Treated by a Nanofibrous Temporary Epidermal Layer

**DOI:** 10.3390/jcm12165273

**Published:** 2023-08-13

**Authors:** Mauro Vasella, Jan Cirebea, Epameinondas Gousopoulos, Anna Wang, Riccardo Schweizer, Matthias Waldner, Gerrit Grieb, Philipp Buehler, Jan Alexander Plock, Bong-Sung Kim

**Affiliations:** 1 Department of Plastic Surgery and Hand Surgery, University Hospital Zurich, 8091 Zurich, Switzerland; mauro.vasella@usz.ch (M.V.); epameinondas.gousopoulos@usz.ch (E.G.); anna.wang@ksa.ch (A.W.); riccardo.schweizer@gmail.com (R.S.); matthias.waldner@usz.ch (M.W.); jan.plock@ksa.ch (J.A.P.); 2 Department of Plastic Surgery and Hand Surgery, Cantonal Hospital Aarau, 5001 Aarau, Switzerland; 3 Department of Plastic, Reconstructive and Aesthetic Surgery, Regional Hospital Lugano, 6900 Lugano, Switzerland; 4 Department of Plastic Surgery and Hand Surgery, Gemeinschaftskrankenhaus Havelhoehe, 14089 Berlin, Germany; gerritchristian.grieb@havelhoehe.de; 5 Department of Plastic Surgery & Hand Surgery, Burn Center, Medical Faculty, Hospital of the RWTH Aachen University, 52074 Aachen, Germany; 6 Center of Intensive Care Medicine, Cantonal Hospital Winterthur, 8400 Winterthur, Switzerland; philipp.buehler@ksw.ch

**Keywords:** facial burns, conservative treatment, nanofibrous dressing, temporary dressing

## Abstract

Background: The face is commonly affected in thermal injuries, with a demand for proper recognition and the correct choice of treatment to guarantee optimal aesthetic and functional outcomes. It is highly vascularized and often heals conservatively, highlighting the particular relevance of conservative treatment modalities, many of which require daily re-applications or dressing changes, which can be painful and tedious for both the patient and the healthcare providers. Motivated by encouraging results of a novel temporary nanofibrous epidermal layer, we herein present a case series of this technology in a case series of patients suffering from facial burns and treated in our Burn Center. Patients and Methods: Patients with superficial partial-thickness facial burns and mixed pattern burns, which were treated with SpinCare^™^, an electrospun nanofibrous temporary epidermal layer, between 2019 and 2021, at our institution were analyzed retrospectively. The Manchester scar scale (MSS) and numeric rating scale (NRS) were used for scar, pain, and outcome evaluation at different time points by five independent board-certified plastic surgeons with profound experience in burn surgery. Results: Ten patients (m = 9; f = 1) were treated and evaluated retrospectively. The mean age was 38.8 ± years (SD ± 17.85). The mean healing time was 6.4 days (SD ± 1.56). The mean follow-up was 16.4 months (SD ± 11.33). The mean MSS score was 5.06 (SD ± 1.31), and the mean NRS Score for pain was significantly reduced from initially 7 to 0.875 upon application (mean (pre-application) 7 ± 0.7 and (application) 0.875 ± 1.26; *p* ≤ 0.0001). Patients reported a NRS score of 10 in terms of functional and cosmetic outcomes at their final follow-up appointment. No adverse effects were observed. Conclusions: The application of a nanofibrous temporary epidermal layer such as SpinCare^™^ represents a relatively easy-to-use, well-tolerated, and effective alternative for the treatment of partial-thickness facial burns.

## 1. Background

Burn injuries involving the facial area are commonly encountered in burn centers. In extensive burns and work-related accidents, the face is involved in over 50% of events, as reported earlier [1,2,3]. Similar to injuries of the hands, the face represents an area of aesthetic and functional importance. Beyond vital tasks of eating, drinking, and breathing, facial features are essential in the patient’s identity and ability to socialize with other people, including non-verbal communication [2,3,4,5,6]. In burn surgery, facial disfigurement due to hypertrophic scarring is a serious concern [3,4]. The face, compared to other areas, is highly vascularized and will often heal conservatively, even in cases with deep partial-thickness injuries [2,4,7,8,9]. Therefore, early excision and skin grafting for partial-thickness burns is a less favored strategy in our experience. While the most crucial qualities of facial wound coverage products are the capability to guide unimpaired wound healing via adequate wound hydration and protection, which prohibits pathological scarring [10], practicability is another, commonly underestimated aspect. Occlusive wound products that require daily painful re-applications or changes, or cause discomfort by inhibiting social reintegration and participation in daily activities, may hamper patient satisfaction and compliance, and potentially worsen the outcome due to premature abandonment of therapy. Another particular challenge in the conservative treatment of facial burn wounds are facial movements, maintenance of vision, and oral hygiene, which is a tremendously demanding endeavor [7,9].

Multiple conservative regimens have been used and described by various authors in the past and include Suprathel^®^ (PolyMedics Innovations GmbH, Denkendorf, Germany), Epicite hydro^®^ (QRSKIN GmbH, Würzburg, Germany), and hyaluronic acid-based dressings, amongst others [10,11,12,13,14].

Recently, Schulz et al. published their experience using a novel electrospun nanofibrous temporary epidermal layer called SpinCare^™^ (Nanomedic Technologies Ltd., Lod, Israel) for the treatment of donor sites and burn wounds of various anatomical areas with promising results [15].

Electrospinning is a versatile and efficient technique used to produce ultrafine fibers, using an electric field to generate a jet of polymer solution or melt that elongates and solidifies into fibers as it travels toward a grounded collector. The process of electrospinning allows for the creation of fibers with diameters ranging from nanometers to a few micrometers. The setup typically consists of a high-voltage power supply, a syringe or spinneret to hold the polymer solution or melt, and a grounded collector. The polymer solution is loaded into the spinneret and a high voltage is applied between the spinneret and the collector. This creates an electric field that induces a charge on the surface of the droplet at the tip of the spinneret. The electrostatic repulsion between the charges leads to the formation of a fine jet of the polymer solution. As the jet travels toward the collector, the solvent evaporates, causing the polymer chains to solidify into fibers [16]. Spincare^™^ promises the application of proprietary nano-diameter fibers via a portable, easy-to-use device [17].

Considering the relative dearth of data with regard to topical treatments of facial burns [2,18], we aimed to evaluate Spincare’s^™^ feasibility based on its reported versatility and analgesic effect in the treatment of facial burns in a case series performed at our Burn Center at the Zurich University Hospital.

## 2. Materials and Methods

### 2.1. Study Design

We retrospectively analyzed the data of patients with facial burns treated at the Burn Center of the University Hospital Zurich between 2019 and 2021. Inclusion criteria were: burns of the facial area with at least 1% total burned surface area (TBSA), superficial partial-thickness burns, or deep partial-thickness burns, and signed informed consent. Exclusion criteria were: patients younger than 16 years of age, isolated superficial or full-thickness burns of the face, pregnancy/nursing, and rejection of informed consent.

All patients were treated with a nanofibrous temporary epidermal layer called SpinCare^™^. The precise content of the utilized material is proprietary. One cartridge was applied, which was loaded in a handheld gun-shaped device. Burn injury and depth were defined on admission by the attending burn surgeon and in accordance with standard guidelines of the American Burn Association criteria [19,20]. The patients were followed up from application until complete wound closure. The local ethics committee provided their consent (BASEC-Nr. Req-2022-00011). All patients approved to participate in the study and publication of anonymized clinical images.

### 2.2. Preparation and Application of SpinCare^™^

All wounds were disinfected with Octenisept^®^ (Schülke & Mayer GmbH, Norderstedt, Germany) or Betadine^®^ (Mundipharma Medical Company, Basel, Switzerland) and debrided prior to Spincare^™^ application. All blisters were removed by sterile gauzes or sponges, and no excision was performed. Additionally, the facial area was shaved with the exception of the eyebrows if necessary. As soon as the face was clean, the orbital and oral areas were protected with either a sterile or paraffin gauze (Jelonet^®^ (Smith & Nephew, London, UK)). Spincare^™^ application was carried out by using the handheld device, which is guided by an incorporated laser that determines the optimal distance of around 20–25 cm from the tip of the device. Application is made through electrospinning, and the patients served as a grounded collector by applying a simple ECG-electrode connected to the handheld device. The optimal thickness of application was achieved when there were signs of a whitish, almost ”frost”-like appearance of the applied nanofibrous layer, which becomes transparent at later stages due to fluid release of the wounds. The treated area was not covered additionally. Forty-eight hours after application, when patients reported sensations of tension in the facial area, a moisturizing ointment (Bepanthen^®^; Bayer AG, Leverkusen, Germany) was applied twice daily. Topical Bepanthen^®^ application was continued for another twelve months.

### 2.3. Outcome Assessment

Photographs of the patient’s facial area were taken at different time points, including on the day of admission, post-debridement, immediately post-application of SpinCare^™^, as well as two and seven days post-intervention, with follow-up at 6 weeks, 3 months, and if possible 6 and 12 months post-application.

Pain was measured by asking the patients to pinpoint a score on a scale between 0 and 10 using the numeric rating scale (NRS) [21] before, and if possible, during the application, as well as 24 h, 48 h, and 7 days post-application, either during hospitalization or in our out-patient department.

The NRS also was used for the assessment of functional and esthetic outcomes, which were rated by the patients individually at their last follow-up consultation in our out-patient department, ranging from 0 to 10, with 0 being the worst functional or aesthetic outcome and 10 being the best possible.

Healing time was assessed by five independent board-certified attending plastic surgeons with an average of 9.4 years of burn surgery experience using photographs taken before treatment, during hospitalization, and regular out-patient visitations, usually at 2, 6, 12, 24, and 52 weeks after treatment if possible. Wound healing was defined as the time point when reepithelization was complete.

Additionally, aesthetic and scar outcomes were assessed using photographs of the last follow-up and the Manchester scar scale (MSS), which is based on the visual analog scale (VAS) of color, finish, contour, distortion, and texture, and ranges from a minimum score of 4 to a maximum of 14 being the worst possible score [22], again evaluated by the same surgeons mentioned above. Details regarding the MSS, its categories, and scoring can be found in Table 1. Data is represented as range and mean ± SD. Statistical analysis for pain scores was performed by one-way ANOVA with Tukey’s multiple comparisons test with GraphPad Prism V8.0 (GraphPad Software, San Diego, CA, USA). *p*  <  0.05 was accepted as statistically significant.

## 3. Results

### 3.1. Data

#### 3.1.1. Patients

A total of 10 patients (9 male, 1 female) were included. The mean age was 38.8 years (range 16–72 years; SD ± 17.85). Two out of ten patients presented mixed pattern burns with small parts of deep partial-thickness burns alongside mostly superficial partial-thickness burns. The other eight patients suffered from superficial partial-thickness burns only. Mean TBSA was 16.5% (range 3.5–35%; SD ± 8.91), and mean facial TBSA was 3.2% (range 1–4%: SD ± 1.059). The mean follow-up was 16.4 months (range 2–31.5 months; SD ± 11.33). Follow-up for patients #7 and #8 was incomplete consultations as we were unable to contact #7, while #8 was deceased of unknown causality after discharge from our Burn Center. A full overview of the patient data, including the measured parameters, can be appreciated in Table 2.

#### 3.1.2. Pain Scores

**Figure 1:**
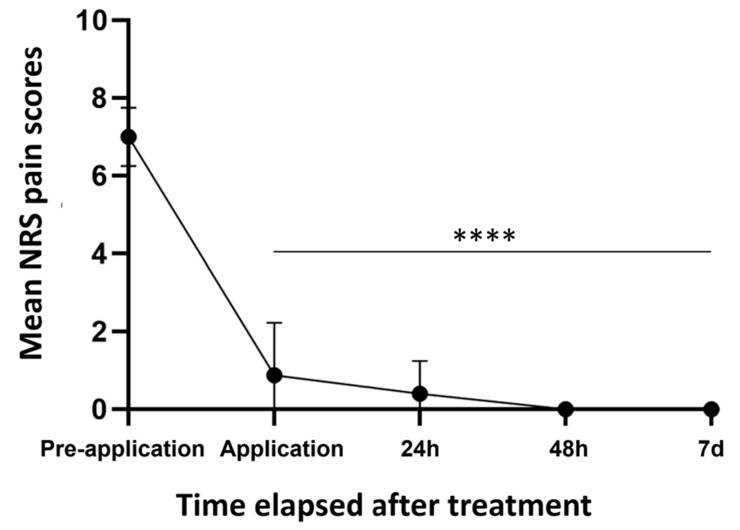
Average NRS score for pain reported by patients before, during, and 24, 48, and 72 h after application of Spincare™ which shows a significant decline in pain sensation already upon application (*p* ≤ 0.0001). **** signifies *p* ≤ 0.0001 when compared to the pre-application score.

All patients reported an analgetic effect upon application with a mean NRS for pain before application of 7 which was significantly reduced to 0.875 during application (mean (pre-application) 7 ± 0.7 and (application) 0.875 ± 1.26; *p* ≤ 0.0001). Pain scores were reduced further to a mean of 0.4 after 24 h; however, the reduction was non-significant when compared to the timepoint of application (mean (application) 0.875 ± 1.26 and (24 h) 0.4 ± 0.8; *p* = 0.3751). A mean score of 0 was recorded after 48 h as well as 7 days post-treatment. The mean pain score over time is depicted in Figure 1. The pain perception of patients #1 and #6 was not evaluated before and during the application, as the patients were already intubated. There was no difference in pain perception when comparing superficial (*n* = 8) and deep partial-thickness injuries (*n* = 2), gender, or different facial areas. Additionally, higher pain scores did not correlate with higher TBSA.

#### 3.1.3. Healing Time

The mean healing time, i.e., time until complete epithelization, was 6.4 days (range 5–10; SD ± 1.56). There was no significant difference in healing time when comparing superficial or deep partial-thickness injuries, as well as when comparing both genders or different facial areas.

#### 3.1.4. Functional and Aesthetic Outcomes

Mean NRS for both functional and aesthetic outcomes were rated by a total of 8 patients with a score of 10 each at their last follow-up, with missing data for patients #7 and #8. None of the patients reported functional limitations or restraining scars, and all of them were content with the aesthetic outcome.

**Figure 2:**
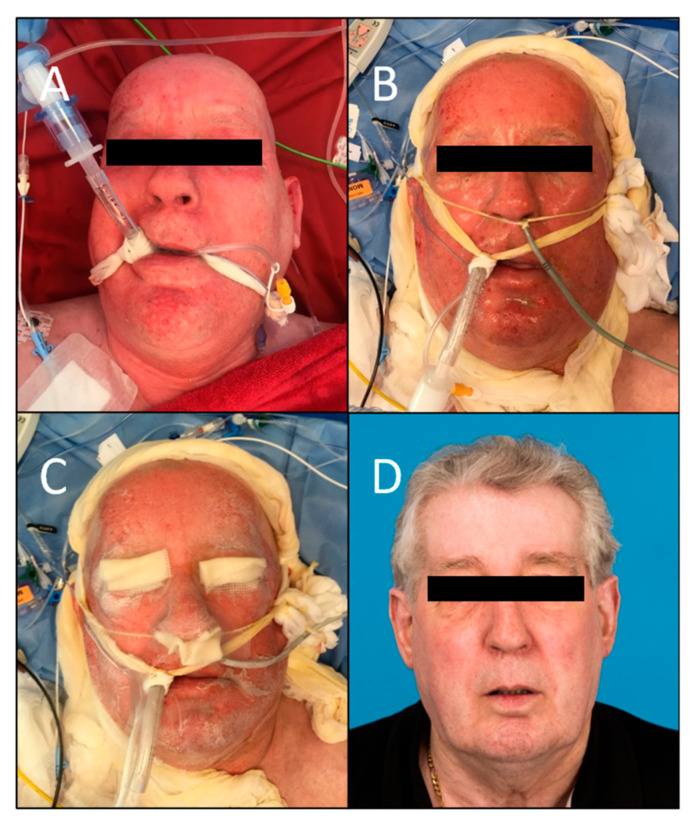
Patient #1 with superficial partial-thickness burns of the face. (**A**) On admission superficial debridement. (**B**) The second day of hospitalization before application of SpinCare^™^. (**C**) After the application of SpinCare^™^. (**D**) Final result 27 months after application.

**Figure 3:**
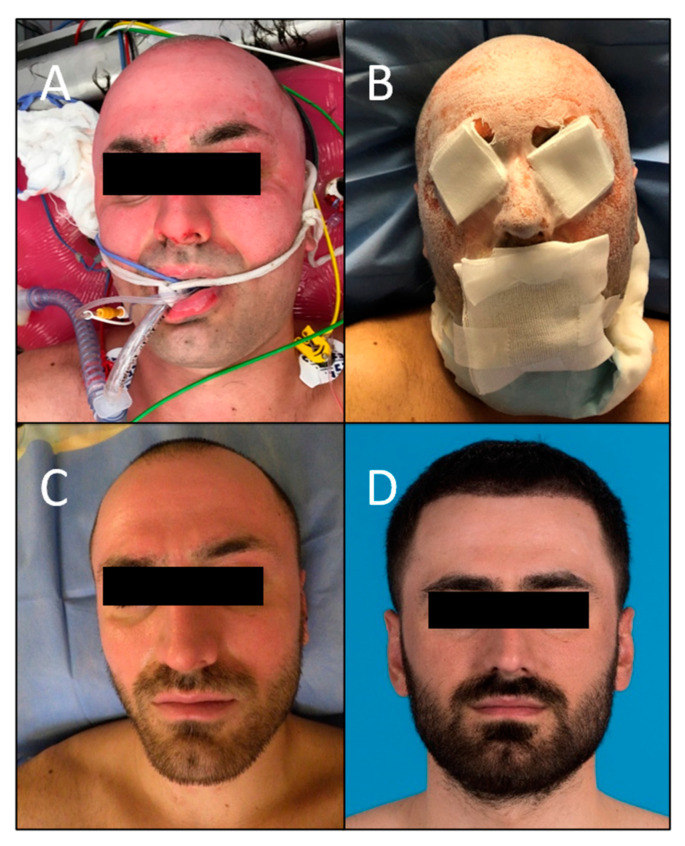
Patient #4 with a mixed pattern burn, including mostly superficial partial-thickness facial burns and parts of deep partial-thickness burns on the forehead. (**A**) On admission after superficial debridement. (**B**) The second day of hospitalization after application of SpinCare^™^. (**C**) One week after application. (**D**) Final result 7.5 months after application.

**Figure 4:**
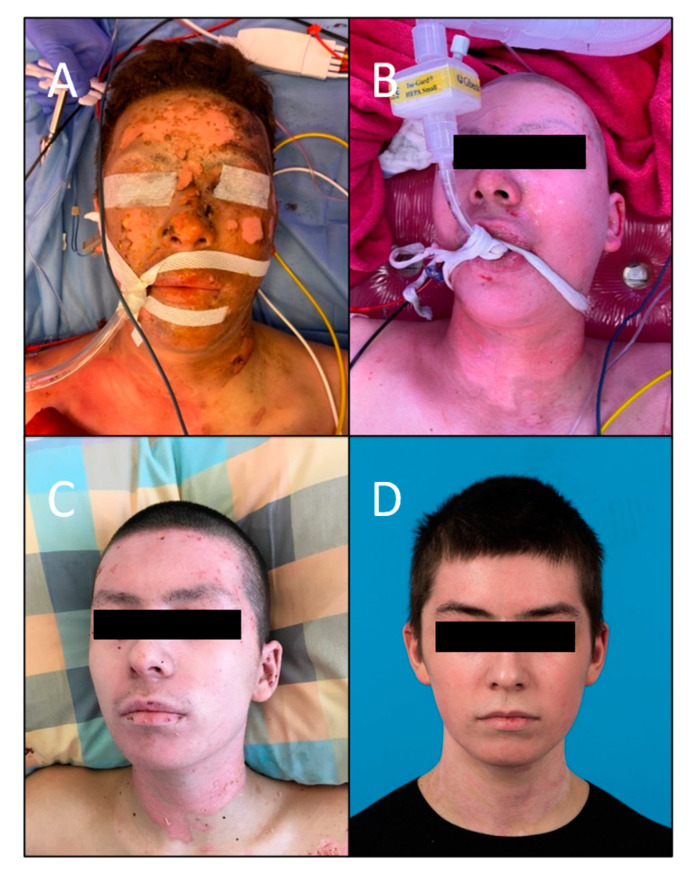
Patient #6 with partial-thickness burns of the face. (**A**) On admission before superficial debridement. (**B**) On admission after superficial debridement. (**C**) One week after the application of SpinCare^™^. (**D**) Final result 18 months after application.

Furthermore, the pictures which were evaluated using the MSS by the board-certified plastic surgeons resulted in a total mean score of 5.06 (range 4–8.8; SD ± 1.31), which is considered low on the VAS ranging from 4 to 14 and, therefore, indicates a positive outcome. This is in line with the subjective scores given by the patients themselves. When breaking the categories down, the mean score for color was 1.64 (range 1–4; SD ± 0.59), 1.12 for finish (range 1–2; SD ± 0.18), 1.22 for contour (range 1–3; SD ± 0.41), and 1.08 for distortion (range 1–3; SD ± 0.24). The mean MSS for every patient can be found in Table 2. The mean scores were similar for the two patients with deep partial-thickness burns when compared to the other patients with superficial partial-thickness burns, and healed well eventually. The initial injury, healing process, and outcome of patients #1, 4, and 6 are demonstrated in Figure 2, Figure 3 and Figure 4.

#### 3.1.5. Application and Complications

No adverse events with regard to the one-time application of Spincare^™^ to the face were reported. No allergic reactions were observed during or after Spincare^™^ treatment, and it was well-tolerated. All wounds healed spontaneously without further intervention. Patients (except patients #7 and #8) reported no difficulties in facial movements, including eating, oral competence, and eye movement.

## 4. Discussion

Given the fact that burn wounds of the face heal spontaneously within an acceptable time frame in the vast majority of superficial partial-thickness burns and even most deep partial-thickness burns, conservative wound management is commonly prioritized over skin grafting [4,23]. A product for optimal conservative treatment of facial burns should satisfy the following needs: fostering wound healing, pain-free application/anesthetic properties, easy adaptability to irregular surfaces, absorbing or permeable to wound fluids, and optimally one-time application process.

Hoogwerf et al. performed a systematic review regarding the different treatment regimens used in facial burns, which incorporated 12 randomized controlled trials (RCTs) and a total of 507 patients [23]. The authors compared studies that investigated topical antimicrobial (e.g., silver sulfadiazine, Aquacel-Ag^™^ (ConvaTec, Braine-l’Alleud, Belgium), cerium-sulfadiazine, gentamicin cream), non-antimicrobial (e.g., saline-soaked dressings, skin substitutes such as allograft or porcine xenograft), and other various treatments (e.g., growth hormone therapy) in burn injuries of the face. They concluded that there is low to very low-certainty evidence that either of these treatments makes a difference in the time of wound healing or in preventing infections. Furthermore, there was only low to very low-certainty evidence regarding the effects that investigated interventions had on patient satisfaction, pain, need for surgery, scar quality, and duration of hospitalization.

However, most of these treatments are, on the one hand, labor-intensive for the care team and, therefore, costly for the healthcare system. On the other hand and more importantly, commonly proposed strategies are rather uncomfortable for the patient due to frequent, in some cases even daily dressing changes/re-application. For instance, products containing silver sulfadiazine are antiseptic but require daily changes, may be painful during the re-application process, and patients should not be exposed to UV light due to the risk of discoloration [24,25]. Other products, such as foam dressings like Mepilex^®^ (Mölnlycke, Gothenburg, Sweden), seem less painful but require regular changes every three to five days, are occlusive, and sometimes antiseptic [26]. Among the variety of products, there are dressings that only necessitate a single application, such as Suprathel^®^, Biobrane^®^ (Smith & Nephew, London, UK), Dressilk^®^ (Prevor, Valmondois, France), or Acticoat7™ (Smith & Nephew, London, UK), and in part have antiseptic and analgetic properties [18,27]. On the downside, all of the aforementioned products have to be molded to the facial silhouette, and sometimes, like Acticoat7^™^, require repetitive moisturizing with sterile distilled water twice a day or on-top occlusive dressing. Also, there is an undeniable risk of dislocation of the aforementioned rigid wound coverages.

For many years, our center has preferred non-occlusive dressings due to obvious advantages such as close wound surveillance, avoidance of regular dressing changes, improved hygiene, and social interaction. In this context, the standard of care for partial-thickness facial burns included the daily application of a prednisone acetate spray. Prednisolone acetate, a synthetic glucocorticoid with anti-inflammatory properties, was used as a moisturizing topical agent in the initial stage of facial burn. The body of literature on the feasibility of topical steroids as a primary treatment regimen in facial burns, however, is sparse. Singer and McClain investigated the effects of high-potency topical steroids in burn wounds in a porcine in vivo model and found that it did not accelerate the re-epithelization [28].

Spincare™ is an alternative non-occlusive dressing with interesting properties. Electrospinning and nanofibrous fibers have attained increased attention over the last decade [29,30]. Electrospun polymer nanofibers are capable of mimicking an extracellular matrix, creating excellent conditions for wound healing which is due to their small pore size, high permeability, and biocompatibility. These characteristics were shown to enhance cellular behavior, for instance, by supporting the migration of keratinocytes [29,30,31,32]. With the help of advanced tissue engineering-based material modifications, polymers may be loaded with drugs to further enhance the regenerative effect [30].

Schulz et al. investigated the application and use of Spincare^™^ in superficial and partial-thickness burn wounds, as well as donor sites, in 10 patients and concluded that Spincare^™^ is a practical and effective treatment [15]. Not only was the healing potential under the said treatment effective in terms of moisture but also adaptable to different surfaces and a one-time application process. The process of Spincare^™^ application was fairly easy in our experience, easily adaptable to the facial contours, and even considered as pleasing as well as analgetic by patients. During application, however, we repetitively noticed electrostatic distortion of the electrospun nanofibers caused by intubation tubes as well as gauzes which would render the application challenging in some cases. Decreasing the application distance for a very short period of time solved the aforementioned issue partially. Once the product was applied, it formed a semipermeable layer that acts as a physical barrier against micro-organisms; however, we believe that it allowed the wound exudate to dry out upon contact with air. The healing time ranged from 5 to 10 days, and none of the patients required a secondary surgery after Spincare^™^ application which is comparable to the study mentioned above as well as the literature reporting a reepithelization ranging from one to two weeks [7,33,34,35,36].

Compared to Schulz et al., who covered the treated area with a silicone layer and dry gauze, we refrained from adding additional dressings immediately after Spincare^™^ application. Instead, a moisturizing cream (Bepanthen^®^) was administered daily after 48 h to moisturize the wounds. By this time, Spincare^™^ mostly had dried out and can be considered as the longevity of the product, which is congruent with Nanomedic’s specification. Aside from the single-stage application, avoiding additional fixation, changes and removal of dressings are a great advantage when compared to the other topical treatments mentioned for facial burns. The downside is that the soft Spincare^™^ layer, particularly in awake patients, is accidentally scraped off by the patient or is unintentionally removed easily during sleep or other daily activities within the first 48 h after application. Also, a strict education of all involved healthcare providers is pivotal to prevent early removal or other untoward topical treatment as the Spincare^™^ layer is hardly noticeable at first sight. The application itself reduced the pain perception significantly, which is in line with Nanomedic’s claim of significantly reducing pain in patients [17]. None of the patients presented with adverse effects, and all of them healed without any hypertrophic scarring or functional disabilities, which, however, would also not be expected with other topical modalities.

This case series must be interpreted with caution, as it primarily serves as an early single-center report of our experience. The nature of this retrospective study with a non-powered sample size and limitation of outcome measures does not allow a more profound recommendation for Spincare^™^. However, after our evaluating our experience and comparing our results with data from the literature, we may assume the treatment of facial burns with Spincare™ is non-inferior when compared to other standard treatment regimens. Also, Nanomedic Technologies Ltd. does not share the formula for its product and therefore prohibits subsequent analysis, mechanistic interpretation, and modification of this technology, which is a substantial downside from a scientific perspective. Aside from the investment for the purchase of the application device, Spincare^™^ was roughly similar in price when compared to the prednisolone acetate treatment.

## 5. Conclusions

Taken together, Spincare^™^ permits a relatively easy, flexible, and analgetic solution for facial burn wounds. While no unfavorable complications were observed, no definite statements regarding the wound healing capacity can be made based on our small series. Nevertheless, its upsides as a non-occlusive dressing make Spincare^™^ an interesting wound coverage candidate for facial burns in the present spectrum of treatment strategies.

## Figures and Tables

**Table 1:** Manchester scar scale employed for the assessment of the outcome of treatment rating color, finish, contour, and distortion.

Parameter		Score
Color	Perfect	1
	Slight mismatch	2
	Obvious mismatch	3
	Gross mismatch	4
Finish	Matte	1
	Shiny	2
Contour	Flush with surrounding skin	1
	Slightly proud/indented	2
	Hypertrophic	3
	Keloid	4
Distortion	None	1
	Mild	2
	Moderate	3
	Severe	4

**Table 2:** Patient demographics. Summarization of patient data including age, gender, burn degree, TBSA total and facial, duration of hospitalization, follow-up, pain scores, scar scores, and functional and aesthetic outcome.

Case	Age	Gender	Burn Degree	TBSA (Facial)	Hospitalization (Days)	Follow Up (Months)	NRS Score Pre-Treatment	NRS Score (24 h, 48 h, 72 h)	Scar Score (MSS)	Functional Outcome	Aesthetic Outcome
1	68	Male	Superficial PT	18 (3.5)	37	27	n/a	2, 0, 0	4.83	10	10
2	42	Male	Superficial PT	11.5 (1)	16	31.5	7	2, 0, 0	4	10	10
3	25	Male	Superficial/deep PT	5.5 (2.5)	12	6.5	6	0, 0, 0	4.8	10	10
4	26	Male	Superficial/deep PT	16.5 (2)	11	7.5	7	0, 0, 0	4	10	10
5	23	Male	Superficial PT	3.5 (3)	5	30	6	0, 0, 0	4.8	10	10
6	16	Male	Superficial PT	24 (4)	25	18	n/a	0, 0, 0	5.4	10	10
7	31	Female	Superficial PT	10.5 (4)	7	3	8	0, 0, 0	5.2	n/a	n/a
8	40	Male	Superficial PT	35 (4)	89	3	7	0, 0, 0	8.8	n/a	n/a
9	45	Male	Superficial PT	17 (4)	26	29	7	0, 0, 0	4.4	10	10
10	72	Male	Superficial PT	23 (4)	22	8.5	8	0, 0, 0	4.6	10	10

## Data Availability

No new data were created or analyzed in this study. Data sharing is not applicable to this article. Restrictions apply to the availability of the formulation of the investigated product due to patent protection.
